# Sinapic Acid Ameliorates Acetic Acid-Induced Ulcerative Colitis in Rats by Suppressing Inflammation, Oxidative Stress, and Apoptosis

**DOI:** 10.3390/molecules27134139

**Published:** 2022-06-28

**Authors:** Mudassar Shahid, Mohammad Raish, Ajaz Ahmad, Yousef A. Bin Jardan, Mushtaq Ahmad Ansari, Abdul Ahad, Khalid M. Alkharfy, Ahmed L. Alaofi, Fahad I. Al-Jenoobi

**Affiliations:** 1 Department of Pharmaceutics, College of Pharmacy, King Saud University, Riyadh 11451, Saudi Arabia; mahmad1@ksu.edu.sa (M.S.); ybinjardan@ksu.edu.sa (Y.A.B.J.); aahad@ksu.edu.sa (A.A.); ahmedofi@ksu.edu.sa (A.L.A.); aljenobi@ksu.edu.sa (F.I.A.-J.); 2 Department of Clinical Pharmacy, College of Pharmacy, King Saud University, Riyadh 11451, Saudi Arabia; aajaz@ksu.edu.sa (A.A.); alkharfy@ksu.edu.sa (K.M.A.); 3 Department of Pharmacology and Toxicology, College of Pharmacy, King Saud University, Riyadh 11451, Saudi Arabia; muansari@ksu.edu.sa

**Keywords:** ulcerative colitis, sinapic acid, acetic acid, inflammatory markers, apoptosis

## Abstract

***Background***: Ulcerative colitis (UC) is a long-term condition which results in inflammation and ulcers of the colon and rectum. The key indications of active disease are abdominal pain and diarrhea mixed with blood. ***Aims***: We explore the underlying colon protective mechanism of sinapic acid (SA) against acetic acid (AA) induced ulcerative colitis in rats. The implications of inflammation, oxidative stress, and apoptosis are studied. ***Methodology***: Twenty-four rats were distributed into four categories, normal control (NC), ulcerative colitis (UC), ulcerative Colitis with SA 40 mg/kg (SA 40 mg/kg + AA), and ulcerative colitis with prednisolone (PRDL 10 mg/kg + AA), and were pretreated orally with saline, saline and SA (40 mg/kg/day) or PRDL (10 mg/kg/day) respectively, for 7 days. UC was prompted by trans-rectal administration of 4% AA on the 5th day, colon tissues were surgically removed for gross morphology and histological inspection, oxidative stress, and inflammatory markers and immunoblot analysis of Bax, caspase-3, and Bcl-2. ***Results***: Macroscopic and histological inspection demonstrated that both SA 40 mg/kg and PRDL (10 mg/kg/day) significantly ameliorates colonic injuries. In addition, both pretreatments significantly ameliorates AA-induced UC, oxidative stress, as indicated by suppressed malondialdehyde (MDA), nitric oxide (NO) levels and restoring antioxidant/oxidant balance as indicated by catalase and glutathione levels, suppressed inflammation via inhibiting cytokines TNF-α, IL-6, inflammatory markers MPO, PGE_2_, COX-2 and NF-κB and inhibiting the protein expression of Bax and caspase-3 apoptotic protein and increasing the anti-apoptotic protein, Bcl-2 thereby inhibiting apoptosis. ***Conclusion***: Sinapic acid significantly ameliorates AA induced UC in rats by suppressing inflammation, oxidative stress, and apoptosis in colonic tissues which exhibits its potential for the management of UC.

## 1. Introduction

Ulcerative colitis (UC) is a persistent inflammation of colonic mucosa and is clinically described by nausea, abdominal pain, loss of weight, and rectal bleeding that affect the wellbeing [1]. The prevalence of idiopathic inflammatory bowel disease (IBD) including UC is substantially high, with an estimation of 25 patients per 10,000 people in the west but in other parts of the world too due to modern lifestyle [2,3]. The etiology of IBD is still not clear, but it is usually admitted that it results from an intricate relationship within environmental, microbial, genetic, and immunogenic factors that activate in mucosal T- cell immune response, which prompts the release of inflammatory mediators such as reactive oxygen species (ROS) and reactive nitrogen species (RNS), reduction of antioxidant enzymes of the colonic mucosa, loss of integrity of the mucosal barrier, anti-inflammatory cytokines and markers, cell infiltrations, and NF-κB activation, which cause apoptotic injuries in the colon [4,5]. Apoptosis has been implicated in the pathophysiology of IBD in previous investigations [6,7,8]. Inflammatory response alter mucosal barrier function and intestinal integrity leading to apoptosis [9]. Inflammatory bowel diseases and UC is a multifactorial disorder without a clear remedy to control oxidative stress and immune response [1]. Still drugs steroids (glucocorticoid), 5-amino-salicylic acid (5-ASA) derivatives, immunosuppressive antibiotics and anti-tumor necrosis factor TNF-α (certolizumab pegol, etanercept) etc. in treatments of IBD and UC [10]. These drugs are generally associated with detrimental effects, with low therapeutic efficacy or no response to treatments [11,12]. Due to rising dissatisfaction with current treatments, there is regime emphasis on natural products such as phytoconstituent with potent antioxidant and anti-inflammatory ameliorate UC with fewer and no side effects.

**Figure 1:**
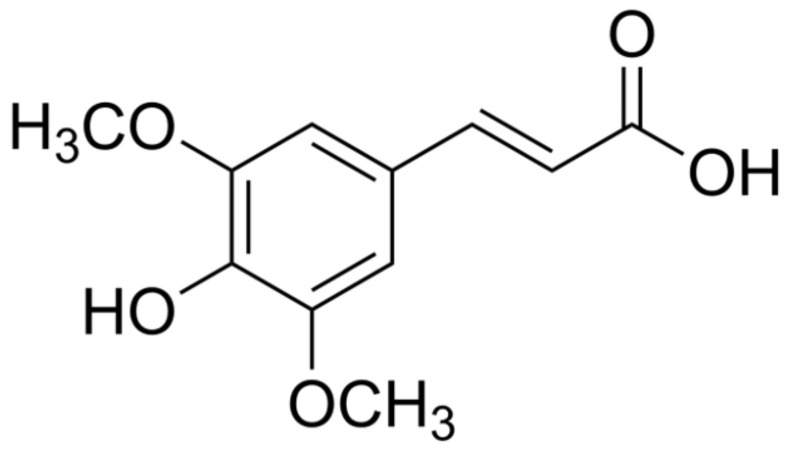
Chemical structure of sinapic acid.

Sinapic Acid (SA) is a polyphenol (3,5-Dimethoxy-4-Hydroxycinnamic acid, Figure 1), the main phytoconstituent plentifully present in plants of family Brassicaceae, citrus berry fruits, vegetables, cereals, and oil-seed crops that are commonly consumed as food and beverages [13]. It is very famous for its potent chemopreventive biological effects, antioxidant, antihypertensive, antidiabetic, anti-inflammatory, anti-anxiety, gastro protective, hepatoprotective, and neuroprotective effects [8,13,14,15,16,17,18,19]. In rodent models, oral or systemic administration of phenolic acids such as epigallocatechin gallate, resveratrol, quercetin, and curcumin downregulate nuclear factor (NF-κ B)-dependent gene expression and upregulate phase II detoxifying and antioxidant proteins. Polyphenols prevented UC [20,21,22,23]. Yet no studies have been performed to assess the protective effect of SA against AA-induced UC in a rat model and mechanisms remain vague. Thus, this investigation aimed to probe the underlying colon protective mechanism of SA AA-induced UC in rats and the implications of inflammation, oxidative stress, and apoptosis.

## 2. Materials and Methods

### 2.1. Drugs and Chemicals

SA, AA, and PRDL from Sigma-Aldrich (St. Louis, MO, USA). MPO, IL-6, and TNF-α, ELISA kits from R&D Systems. (Minneapolis, MN, USA). NE-PER and Pierce™ BCA Protein Assay Kit Thermo fisher Scientific™ (Waltham, MA, USA). The primary antibodies from Santa Cruz Biotechnology (Dallas, TX, USA): Caspase 3, Apoptosis Regulator (Bax), B-cell lymphoma 2 (Bcl-2), nuclear factor kappa-light-chain-enhancer of activated B cells (NF-κB), Cyclo-oxygenase-2 (COX-2), Heme oxygenase (HO-1), Nuclear factor E2-related factor 2 (Nrf2), beta-actin (β-actin) and HRP-conjugated secondary antibodies.

### 2.2. Animals

Male Wistar rats (10–12 weeks old, weighing 206–227 g) were procured from the animal facility of King Saud University, Riyadh, Saudi Arabia. They were kept under standard conditions 24 ± 2 °C, 12 h light/dark cycle, and received water and food ad libitum. The Ethics Committee of the Experimental Animal Care Society, King Saud University, Saudi Arabia [KSU-SE-21-59], approved the experimental plan. All animals were treated as per the guidelines of the National Institute of Health Guidelines for the Care and Use of Laboratory Animals.

### 2.3. Experimental Design

Animals were indiscriminately allocated into 4 categories (6 rats/group); Normal control; UC control; SA treated group: rats received SA (40 mg/kg, orally) and PDRL treated group: rats received PDRL (10 mg/kg, orally). Before induction of UC, rats were given either the normal saline or the treatment for 7 days in a row, with the last dosage of either the normal saline or the treatment given one hour before induction of UC. Concisely, on day seven, the animals were weighed and provided with water ad libitum, but fasted for 12 h. The animals were anesthetized with xylazine and ketamine (5 mg/kg, i.p.). In all groups, an enema was conducted by instilling 2 mL of saline by a feeding gavage 4.5 cm from the rectum. A 20 mL syringe linked to feeding gavage was used to aspirate the fecal content.

### 2.4. Induction of Colitis

UC was prompted by the intrarectal administration of 2 mL of 4 % acetic acid (AA) by a feeding gavage (2 mm in diameter), which was introduced to a depth of 4.5 cm inside the rectum. To prevent intracolonic solution leakage, the rats were held in the inverted Trendelenburg position during rectal installation and for two min thereafter, euthanized in a CO_2_ chamber [24,25].

### 2.5. Assessments of Colitis

#### 2.5.1. Assessment of Colon Weight-to-Length Ratio

The distal 10 cm colon was excised, opened lengthwise, and washed in normal saline to remove the feces. Colons were placed on non-absorbent surfaces and colon weight-to-length ratios were indiscriminately evaluated.

#### 2.5.2. Macroscopic Scoring

Damage scores were allocated based on gross macroscopical features of the colon using a scoring pattern of Morris et al., 1989 [26]. Nonpartisan examiner unknown to the treatment assessed the sternness of colitis. After gross examination, the colon was cut into four pieces and stored at −80 °C and one fragment fixed in 10% formalin (pH 7.4) for 48 h for histopathological analysis.

#### 2.5.3. Evaluation of the Disease Activity, Ulcer Index and Protection Index

Disease activity index (DAI) was calculated to check the sternness of colitis by employing scoring scale for weight loss, diarrhea, and hematochezia (Table 1).

Colitis induced by AA is evaluated by the DAI [27]. The ulcer area and ulcer index were examined as per Dengiz and Gursan [28]. Using the following formula, the ulcer index was calculated:

Ulcer index (UI) = total ulcer area (mm^2^) ÷ Total area of colon (mm^2^)

$$\mathrm{Protection}\;\mathrm{index}\;\left(\mathrm{PI}\right)=\left[\frac{\mathrm{UI}\;\mathrm{control}-\mathrm{UI}\;\mathrm{treated}}{\mathrm{UI}\;\mathrm{control}\;}\right]\times 100$$

### 2.6. Cytosolic and Nuclear Protein Extracts Preparations

The quantification of total protein was performed using Bicinchoninic acid (BCA) total protein assay kits was employed to quantify total protein as per the manufacturer’s instructions [29]. The nuclear and cytoplasmic isolation was performed on colon tissues using an NE-PER Thermo fisher Scientific, ™ USA as per to the manufacturer’s instruction.

### 2.7. Oxidative Stress and Antioxidant Enzyme Indices

Lipid Peroxidation (MDA) Assay and nitric oxide (NO) content were estimated in colon tissues using a colorimetric assay kit (Abcam, Cambridge, UK). A Catalase and Glutathione Colorimetric Activity Kit (Thermo fisher Scientific, ™ USA) was employed to assess the level of Catalase (CAT) and Glutathione (GSH) in homogenized colon tissues as per the manufacturer’s instruction.

### 2.8. Cytokine and Inflammatory Marker

TNF-α, and IL-6 cytokines and PGE_2_ and MPO inflammatory markers levels in colon tissue were quantify by ELISA kits (R&D Systems, Inc., Minneapolis, MN, USA) as per the manufacturer’s protocol and absorbance was scanned at 450 nm.

### 2.9. Western Blot Analysis

Western blot analysis was used to determine protein expression using the protocol of Towbin et al. 1979 [30]. Precisely, 30 g of protein was loaded onto SDS-PAGE gels, electrophoretically transferred to PVDF membranes, blocked with 4% skimmed milk and BSA in TBS containing 1% Tween-20 (TBST), then incubated with primary antibodies overnight at 4 °C. The next day, after five TBST washes, the membranes were incubated with secondary antibodies for 2 h at room temperature. Luminata TM Western Chemiluminescent HRP Substrates were used to visualize the bands (Millipore, Billerica, MA, USA). A LI-COR C-Di-Git Blot Scanner was being used to accomplish a densitometric evaluation of the immunoblots. (Lincoln, NE, USA).

### 2.10. Statistical Analysis

All results are given as mean standard error of the mean (SEM) for every experimental group of *n* = 6 rats. Dunnett’s multiple comparison test or one-way analysis of variance (ANOVA) were used to determine the significance of the sample; *p* < 0.05 was considered significant. Graph Pad V6 statistical software was used in the statistical analysis (GraphPad Software, San Diego, CA, USA).

## 3. Results

### 3.1. Effects of SA on Macroscopic Examination

**Figure 2:**
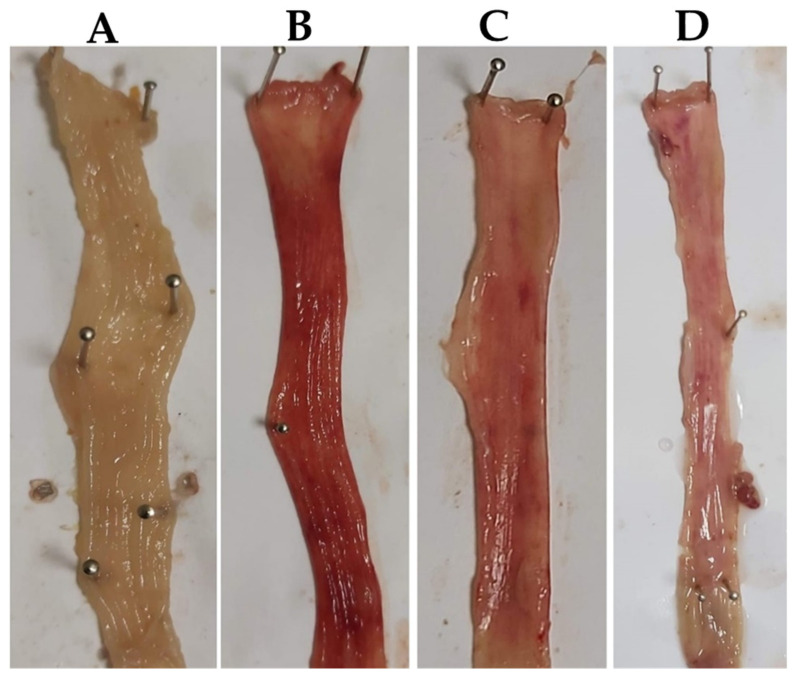
The Gross morphological depiction of colons. (**A**) Normal control, (**B**) acetic acid-treated, (**C**) SA (40 mg/kg)-treated and (**D**) PDRL (2 mg/kg)-treated rats.

The degree and harshness of the colitis triggered by AA administration were assessed in all groups. Mucosal erosion, mucosal necrosis, ulceration, bleeding, and inflammation, which are the main attributes of UC, were witnessed after intra-rectal 4% AA administration (Figure 2) induced Mucosal erosion, mucosal necrosis, ulceration, bleeding, and inflammation in the rats’ colons. SA (40 mg/kg) and PDRL suppressed AA-induced mucosal erosion, mucosal necrosis, ulceration, bleeding, and inflammation (Figure 2) compared to normal control rats. However, NC rats do not exhibit any sign of ulceration and inflammation. DAI score is indicator of colonic injury and calculated from the symptoms like reduction in body weight, diarrhea and hematochezia. The UC animals indicated high DAI score with symptoms of UC compared to NC. However, SA (40 mg/kg) and PDRL treatments to rats significantly attenuated the increased DAI score (*p* < 0.01) less DAI score (Table 1).

### 3.2. Effect of SA on the Colitis Activity Index

There is significant increase in DAI score, colon weight ratio and decrease in Mucous content in AA induced ulcerative colitis animal compared to controls. SA (40 mg/kg) and PDRL treatment ameliorated the AA effect significantly. However, regarding the colon weight, SA 40 mg/kg and PRDL 10 mg/kg treatments significantly counterbalance the increase in colon weight and decrease in mucus content. AA-induced colitis rats showed a significant increase in body weight loss, as displayed in Table 2. However, SA- and PDRL-treated UC rats exhibited a decline in body weight loss compared to UC rats.

### 3.3. Effect of SA on Oxidative Stress

**Figure 3:**
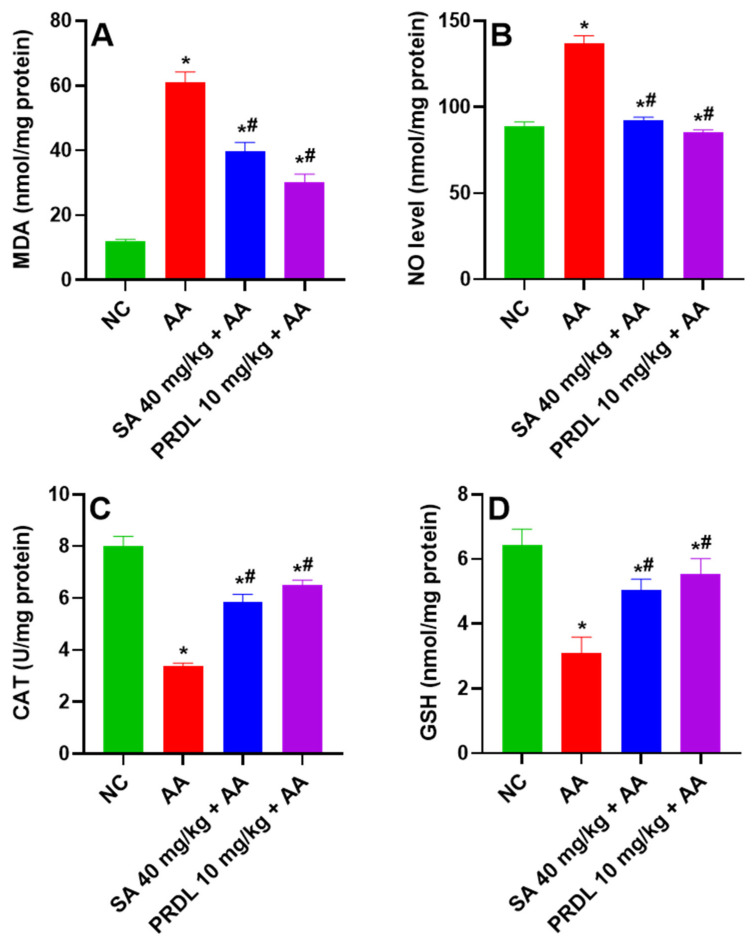
Levels of (**A**) MDA, (**B**) NO, (**C**) CAT and (**D**) GSH in in AA-induced ulcerative colitis rats. The results are presented as mean ± SEM (*n* = 6) per group. * denotes significant differences compared to the control group (*p* < 0.05); # denotes significant differences compared to the AA group (*p* < 0.05).

Figure 3A,B represent the effect of SA on lipid peroxidation and nitrosative stress in colon. AA-induced UC animal’s demonstrated significant escalation in lipid peroxidation (MDA) and nitrosative stress (NO) revealing enhanced oxidative stress as compared to NC. Animals treated with SA 40 mg/kg and PDRL 10 mg/kg demonstrated a significant decrease in mean MDA level and NO compared to UC Animals.

### 3.4. Effect of SA on Antioxidant Activity

Figure 3C,D represented the effect of SA on catalase (CAT) and glutathione (GSH) antioxidant activity in colon tissue of all animals. AA-induced UC animals demonstrated significant depletions in CAT and GSH levels, revealing a depletion in free radical scavenging activity compared to NCs. Animals treated with SA 40 mg/kg and PDRL 10 mg/kg demonstrated significant replenishment in mean CAT and GSH levels compared UC animals, indicating restoration of free radical scavenging activity.

### 3.5. Effect of SA on Inflammatory Cytokines

**Figure 4:**
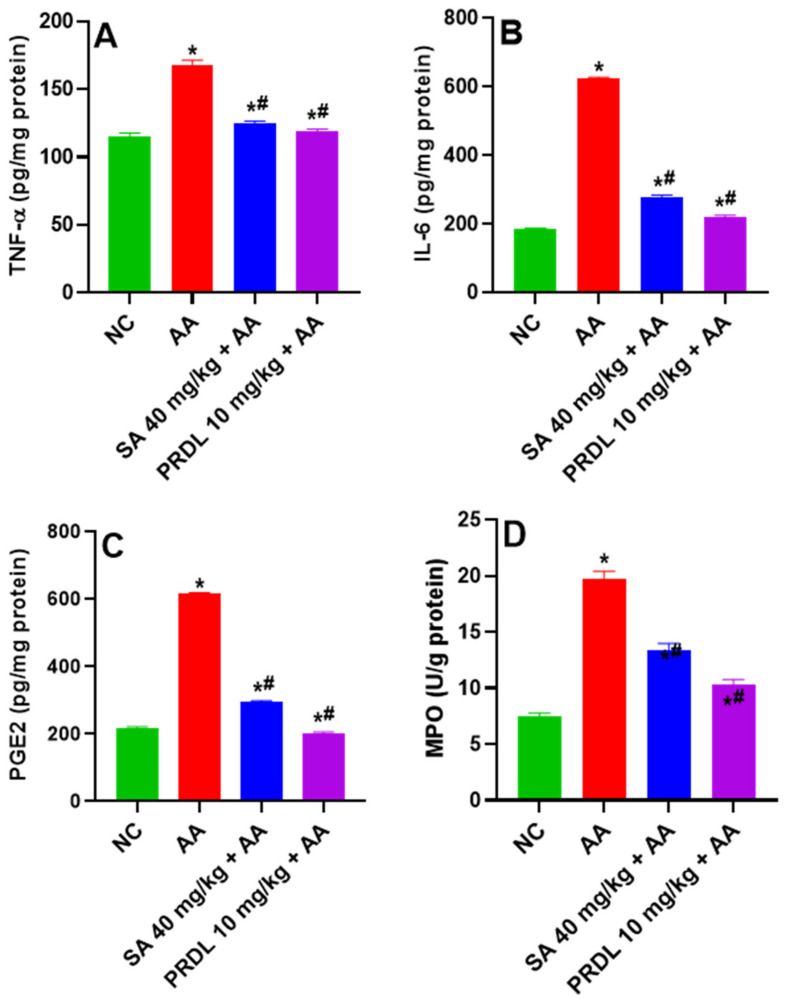
Levels of (**A**) TNF-α, (**B**) IL-6, (**C**) PGE_2_ and (**D**) MPO levels in AA-induced ulcerative colitis rats. The results are presented as mean ± SEM (*n* = 6) per group. * denotes significant differences compared to the control group (*p* < 0.05); # denotes significant differences compared to the AA group (*p* < 0.05).

Figure 4A,B represents the effect of SA on cytokines TNF-α and IL-6 in colon tissue of all animals. AA-induced UC animals demonstrated significant augmentations in cytokines TNF-α and IL-6, revealing enhanced inflammation in the colon as compared to NC. However, animals treated with SA 40 mg/kg and PDRL 10 mg/kg demonstrated a significant decline in mean TNF-α and IL-6 compared to UC Animals.

### 3.6. Effect of SA on Inflammatory Markers

Figure 4C,D represented the effect of SA on inflammatory marker PGE_2_ and MPO in colon tissue of all animals. PGE_2_ and MPO play a key role in the UC. The observed results exhibited significantly (*p <* 0.001) upregulation of PGE_2_ and MPO levels in AA-induced UC compared to NCs. SA 40 mg/kg and PDRL treatment significantly reduced this upregulated concentration of PGE_2_ and MPO as compared to ulcerative colitis, as shown in Figure 4C,D.

### 3.7. SA Downregulates Apoptosis, COX-2, iNOS and NF-κB (p65) in Ulcerative Colitis

**Figure 5:**
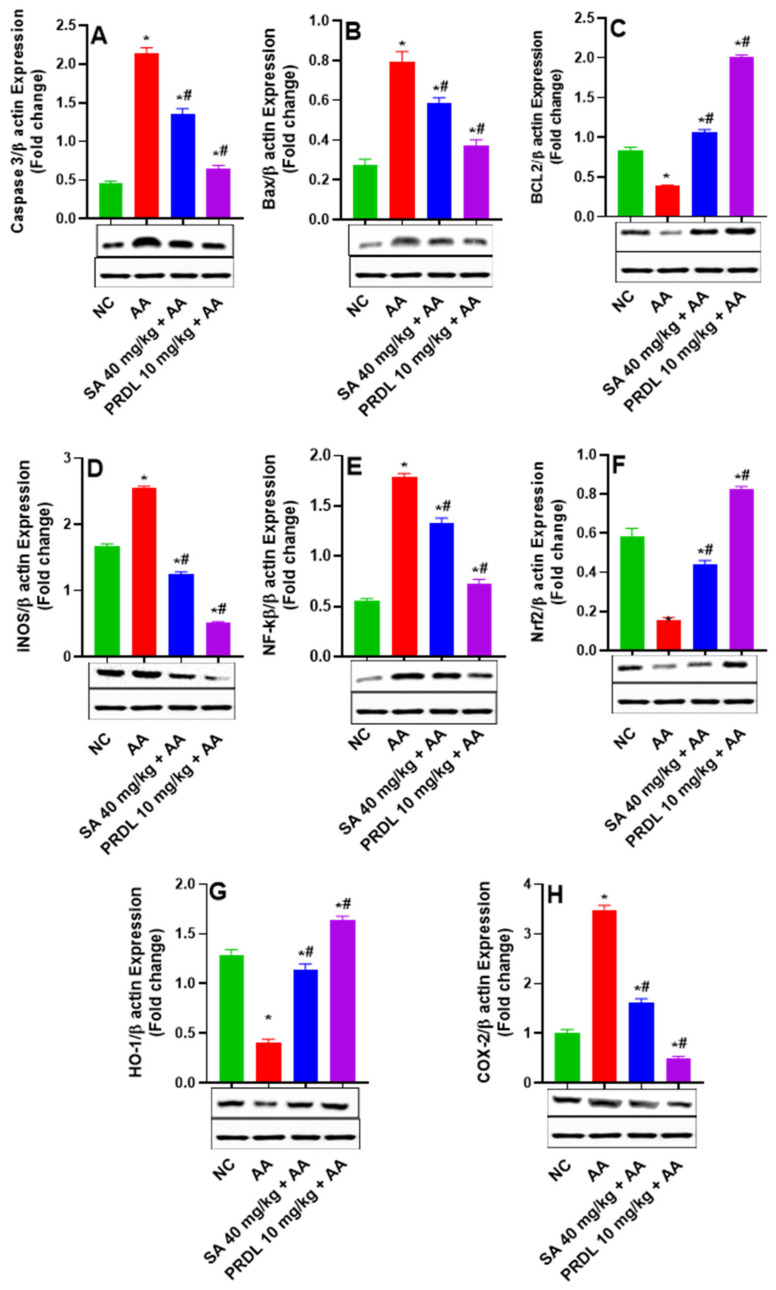
Effects of SA on apoptotic proteins and restoration of antioxidant defense (**A**) Caspase 3, (**B**) Bax, (**C**) BCl_2_ (**D**) iNOS, (**E**) NF-kB (p65 heterodimer), (**F**) NRF2, (**G**) HO-1 and (**H**) COX-2 expression levels in AA-induced ulcerative colitis rats. The protein expression was analyzed in colon tissue of all experiment groups. The results are presented as mean ± SEM (*n* = 6) per group. * denotes significant differences compared to the control group (*p* < 0.05); # denotes significant differences compared to the AA group (*p* < 0.05).

As demonstrated in Figure 5, AA prompted UC colon tissues exhibits upregulation in the protein expression of proapoptotic proteins caspase-3, BAX and downregulation in the expression of antiapoptotic proteins Bcl-2, which demonstrate induction of apoptosis in colonic mucosa. However, SA 40 mg/kg and PDRL treatment significantly ameliorates the AA prompted apoptotic injuries evident by significant downregulation in the protein expression of proapoptotic proteins caspase-3, BAX and upregulation of Bcl-2 was observed. These results indicate that SA inhibited AA-induced colon epithelial apoptosis by affecting the expression of proapoptotic-related factors and induction of antiapoptotic factors. The activity of 40 mg/kg SA and 10 mg/kg PDRL on NF-κB activation, as demonstrated in Figure 5, the protein expression of NF-κB significantly augmented in the AA-prompted UC group, relative to the NC. Yet, 40 mg/kg treatment with SA and PDRL 10 mg/kg reduced NF-κB (p65) expression significantly. The data demonstrated that SA and PDRL might inhibit AA-prompted UC by inhibiting NF-κB activation and stimulated the translocation of p65 into the nucleus, indicating inhibition of apoptosis and inflammations. Furthermore, the AA-induced UC colonic mucosa significantly enhanced the iNOS protein expression and 40 mg/kg SA and 10 mg/kg PDRL treatment significantly suppressed iNOS protein expression as compared to AA-prompted UC. Indicating potent anti-inflammatory effect. The AA-treated colonic tissue demonstrated significant upregulation of COX-2 protein; however, SA 40 mg/kg and PDRL 10 mg/kg significantly downregulated the COX-2 expression colitis tissue. The AA-prompted UC colonic tissues demonstrated significant suppression of Nrf2 and HO-1 protein expression compared to NCs. Conversely 40 mg/kg SA and 10 mg/kg PDRL treatment significantly upregulate Nrf2 and HO-1 protein expression compared to AA prompted UC. This indicates the restoration of antioxidant defense mediated by Nrf2 and HO-1 pathways.

### 3.8. Effect of SA on the Histopathological Alteration in AA-Induced Colitis

**Figure 6:**
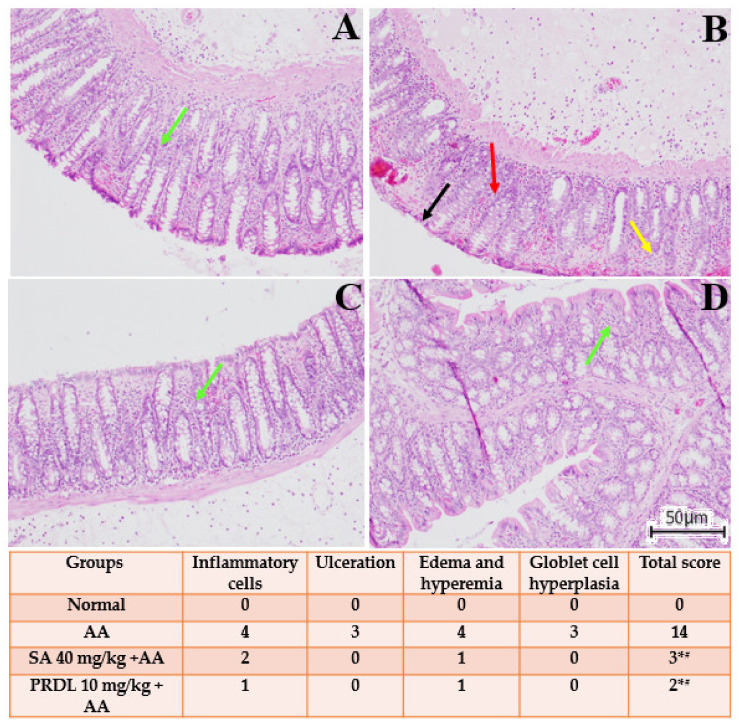
Histopathological observations along with their intensity. Histological colonic sections of a normal rat showing normal mucosa (green arrow) (**A**). Animals in AA group showing active colitis with stromal edema, superficial erosions (black arrow), inflammatory cell infiltration (yellow arrow) with broadly ulcerating mucosa (red arrow) (**B**). SA 40 mg/kg + AA group animals showing ameliorative epithelial changes with ulcer healing and inflammatory cells along with lymphoid follicle (green arrow) (**C**). Animals in (PDRL 10 mg/kg + AA) group exhibiting healing ulcer and attenuation of cell damage (green arrow) (**D**). The results are presented as mean ± SEM (*n* = 6) per group. * denotes significant differences compared to the control group (*p* < 0.05); # denotes significant differences compared to the AA group (*p* < 0.05).

The histological investigation is illustrated in Figure 6, where AA prompted UC colonic mucosa demonstrate transmural necrosis, diffuse inflammatory cell infiltration and erosion of epithelial mucosa. There was ulceration of the colonic mucosa extending through the muscularis mucosa, with desquamated areas. Cryptic architecture was distorted, the lamina propria was thickened in basal areas and with increased infiltration of inflammatory cells was perceived. Treatment with SA 40 mg/kg and PDRL 10 mg/kg significantly attenuated the extent and severity of the histological features of cell damage and restored the architect of colonic mucosa.

## 4. Discussion

UC is a chronic reverting IBD that negatively influences patients’ physical and mental well-being and may increase their risk of colorectal cancer [31]. Anti-inflammatory drugs, corticosteroids, immunosuppresses, biological agents, sulfasalazine and amino salicylates are being used to treat UC symptoms and reduce inflammation. However, there are some adverse effects of these drugs. Thus, studies are needed to develop safer, more effective therapies for UC. The present study examined how SA protected against AA-induced UC. The AA-induced colitis rat model is a well-demonstrated rodent model that shares several signs and symptoms with human ulcerative colitis, comprising severe diarrhea, bloody stools, melena, colonic shortening, mucosal ulcerations, inflammatory cell infiltration, and weight loss [32]. AA induced ulcerative colitis in the colon due to release of protons to the epithelium, thereby causing intracellular epithelial acidification that causes massive epithelial injuries [33,34]. The harshness of the colitis elicited by AA in the colon was examined in all of the animals. The key features of UC, such as mucosal erosion, mucosal necrosis, ulceration, inflammation and bleeding, were observed after induction of UC and per oral treatments of SA 40 mg/kg and PDRL 10 mg/kg repressed the main features of UC (Figure 1) as compared to NC rats. However, normal saline-treated normal rats do not exhibit any signs of ulceration and inflammation. These results are similar to previous findings where polyphenols ameliorate AA-induced ulcerative colitis [22,35,36]. The AA-induced UC escalate in the occurrences of diarrhea, colon weight, that were correlated with inflammation and ulceration. Similar impairments were formerly reported in the rodent models of UC [24]. SA 40 mg/kg and PDRL ameliorated the AA induced impairment as indicated decreased in reduction in body weight, DAI Score, colon weight ratio, ulceration and increased in mucosal content as displayed in Table 1. Treatment with SA 40 mg/kg and PDRL resulted in significant protection. The mechanism by which SA is protected against UC not defined, it may be due to SA inhibits the release or synthesis of inflammatory mediators generated by AA or anti-inflammatory activity/increased mucous content or inhibition of apoptotic injuries. It has been proposed that polyphenols may have ameliorative in disorders associated with oxidative stress and inflammatory diseases [21,34,35,36]. The key contributory factors of IBD are oxidative stress, and depleted free radical scavenging mechanism that controls the generation of ROS and iNOS [7]. The augmentation of ROS and iNOS results in depleted catalase and glutathione antioxidants, which initiated reactive oxygen metabolite cascade and leads to lipid peroxidation (LPO) [2]. Increased in oxidative stress was witnessed in AA induced UC animals confirmed by increased lipid peroxidation in terms of MDA and NO levels indicative of increased ROS and iNOS and depletion of antioxidants enzymes CAT and GSH levels in colon tissues. The SA 40 mg/kg and PDRL pretreatment significantly protect colon mucosal injuries by reducing MDA and NO level as well as significantly and replenished the GSH and CAT levels in colon tissues in AA-induced UC rats. Findings from previous literature are supported by these results [4,22,34,35,37].

AA-induced colitis caused due to widespread intracellular acidification causes erosion of mucosal barriers that ultimately damage the colonic epithelium, which induce activation of inflammatory cytokines releasing cells monocytes and macrophages. The inflammatory cytokines (TNF-α and IL-6) release several chemokines and promotes chemotaxis [36]. SA significantly reduced the elevated level of the inflammatory cytokines (TNF-α and IL-6) thus exhibiting a potent anti-inflammatory effect; our previous reports further corroborate these findings [8,18]. In addition to TNF-α role in enhanced endothelial cell permeability, decreased pain threshold, leukocyte generation, and enhanced prostaglandin (PGEs) levels, in the gastrointestinal epithelium of gastric mucosal tissue, that is a paracrine fashion [38]. In rodent models of AA induced intestinal inflammation, MPO and PGEs levels offer a quantifiable measure of disease severity [39]. MPO and PGEs levels in the inflamed colon were estimated in our study. The pretreatment of SA 40 mg/kg and PDRL medication was able to reduce MPO and PGEs levels in dose-dependent fashion, which may be interpreted as a manifestation of the test compound’s anti-inflammatory properties in the AA model [40].

Cyclooxygenase-2 (COX2) produces PGE2 and thromboxane B2, which stimulate edema and intestinal hyperemia [41]. The activation and upregulation Cyclooxygenase-2 (COX2), PGE2 and iNOS, induced inflammation edema and intestinal hyperemia that leads to augmentation of ROS/RNS and depleted the antioxidative system that induced apoptotic injuries to the colon epithelium [41,42,43]. The protein expression data of AA-induced ulcerative colitis demonstrate upregulation Cox-2, PGE2 and iNOS protein expression that leads to inflammation edema and intestinal hyperemia of the colon epithelium as compared to the normal colon; however, SA and PDRL pretreatment significantly suppressed the Cox-2, PGE2 and iNOS overexpression, probably due to suppression of ROS/RNS and restoration of antioxidative system and suppression of cytokines (TNF α, IL 6 and MPO), apoptotic injuries to the colon epithelium. These results are in line with previous literature where polyphenols inhibit ulcerative colitis as evident by reduced DAI score and intestinal inflammation [36,44,45]. Phenolic acids can inhibit NF-κB, COX-2, iNOS and PGE2, which demonstrate suppression of intestinal edema and amelioration of ulcerative colitis. Phenolic compounds can inhibit NO and PGE2, which results in the suppression of edema [36,45,46,47,48].

The transcription factor NF-κB in its inactive state combined with IκB and activation of NF-κB is dependent on oxidative stress induced by free radicals (ROS, RNS) [49]. The active NF-κB subunits are located in the nucleus, whose primary function involves regulation of inflammatory genes, and it is known as key regulator of the immunological process of IBD [50]. The previous reports implicated an enhanced level of NF-κB is associated with increased levels of oxidative stress and inflammatory cytokines that are causes of increased colon epithelial injuries, leading to colitis [51,52]. In the present investigation, AA induced a significant escalation in levels of NF-κB, TNF-α, IL-6, and oxidative stress and depletion of GSH and CAT in AA-induced colon tissues. These findings further corroborate and reinforce studies that claim that AA aggravates up-regulation of NF-κB and inflammatory cytokines levels in an experimental colitis model in rats [8,23,37]. In contrast, SA 40 mg/kg pretreatment significantly and abridged these elevated levels [18,37,50].

Apoptosis has been implicated in the pathophysiology of IBD in previous investigations [6,7,18,53]. Inflammatory response alters mucosal barrier function and intestinal integrity, leading to apoptosis [9]. In the present study, western blot analysis caspase-3, Bax, and Bcl-2 proteins expressions in colon tissues of AA-induced colitis animals shown a significant increase in protein expression of pro-apoptotic Bax and caspase-3 and suppression of antiapoptotic protein within the intestinal crypts. The SA 40 mg/kg and PDRL 10 mg/kg pretreatment reduced the upregulated pro-apoptotic Bax, caspase-3, and upregulate the antiapoptotic protein Bcl-2, therefore preventing the apoptotic injuries and maintaining the intestinal integrity and mucosal barriers functions. These results further corroborate previous finding where SA pretreatment significantly prevent apoptotic injuries and maintaining the intestinal integrity and mucosal barriers functions [8,14,17,18,54,55]. The microscopical investigation of H&E-stained colon specimens, AA-induced colon epithelial damage in the colitis group and, therefore, raised histological scoring of ulcerative lesions were significantly lessened in SA 40 mg/kg pretreated rats as well as in prednisolone-pretreated rats. Altogether, these findings support the hypothesis that SA 40 mg/kg and PDRL compared AA induced UC rats, can prevent cellular injury and weaken clinical indexes instigated by AA-induced UC in rats.

## 5. Conclusions

In conclusion, Sinapic acid significantly ameliorates acetic acid-induced ulcerative colitis in rats by suppressing inflammation, oxidative stress, and apoptosis in colonic tissues. Therefore, SA has a potential anti-ulcerative colitis effect for the management of UC.

## Figures and Tables

**Table 1:** Scoring of disease activity index.

Animal Groups	Body Weight (g)	% Reduction in BW	Stool Consistency	Rectal Bleeding	DAI Score
Normal	204.17 ± 2.85	0.00	Normal	Normal	0.00 ± 0.00
AA	172.67 ± 2.97	15.43	Bloody diarrhea	Occult +++	3.79 ± 0.10
SA 40 mg/kg + AA	186.50 ± 1.50	8.65	Loose stool	Occult +	1.62 ± 0.05
PRDL 10 mg/kg + AA	190.83 ± 0.95	6.53	Loose stool	Occult +	± 0.06
**Score**	**Body weight decrease (%)**		**Stool consistency**		**Rectal bleeding**
0	<1		Normal		Normal
1	1–5				
2	5–10		Loose stools		
3	10–20				
4	>20		Diarrhea		Gross bleeding

**Table 2:** Effects of SA and PDRL on the macroscopic parameters of ulcerative colitis induced by acetic acid in rats.

Animal Groups	Ulcer Area (mm^2^) (%)	Ulcer Index	Protection Index	Protection of Ulcer	Colon Weight Ratio mg/cm	Mucouse µg/g	Macroscopical Score
Normal	0 ± 0	0.00 ± 0.00	0 ± 0.00	100.00 ± 0.00	78.05 ± 4.45	1506.17 ± 36.78	0.33 ± 0.21
AA	40.52 ± 0.87	0.67 ± 0.04	100.00 ± 3.03	0 ± 3.71	147.23 ± 7.61	541.17 ± 21.15	3.33 ± 0.21
SA 40 mg/kg + AA	13.69 ± 0.71	0.16 ± 0.09	23.70 ± 1.46	76.30 ± 1.79	139.12 ± 4.48	875.67 ± 31.94	1.50 ± 0.22
PRDL 10 mg/kg + AA	6.03 ± 0.40	0.06 ± 0.06	9.46 ± 0.67	90.54 ± 0.82	99.42 ± 2.98	1087.50 ± 31.58	1.33 ± 0.21

## Data Availability

The data generated from the study are clearly presented and discussed in the manuscript.

## Sample Availability

Samples of the compounds are not available from the authors.

## Abbreviations

AA: Acetic; BAX: bcl-2-like protein; Bcl-2: B-cell lymphoma 2; CAT: Catalase; COX-2: Cyclo-oxygenase-2; DAI: Disease activity index; GSH: Glutathione; IBD: inflammatory bowel disease; IL: interleukins; MPO: Myeloperoxidase; MDA: malondialdehyde; NF-κB: nuclear factor kappa-light-chain-enhancer of activated B cells; NO: nitric oxide; NC: normal control; PGE_2_: prostaglandin E2; PRDL: prednisolone; ROS: reactive oxygen species; RNS: reactive nitrogen species; SA: sinapic acid; TNF-α: Tumor necrosis factor-alpha; UC: ulcerative colitis.
